# For Chronic Rheumatism

**Published:** 1887-01

**Authors:** 


For Chronic Rheumatism.—

| | | |
|---|---|---|
| ℞ | Potassium iodide, - - - - | ℥ j |
| | Tr. guiaci. ammo., - - - | fl℥ iij |
| | Ext. phytolacca fl., - - - | flʒ iij |
| | Ext. colchici. sem fl., - - - | flʒ iv |
| | Elixr aurantii q. s. ad., - - - | fl℥ vj. |

---

Among the numerous remedies recommended for *sore nipples*, Prof. Parvin pronounces the compound tincture of benzoin the best, as a local application. As the saliva of the infant is liable to be productive of fissures, etc., by its irritation, the nipple should always be carefully cleansed and dried after the nursing of the child.

---

The birth-rate in Buffalo during 1886 was 12 per 1,000 in excess of the death-rate.

M. De Quatrefages exhibited at a meeting of the French Academy of Sciences, two skulls discovered in a cave in Belgium. These skulls belong to the quaternary geological period, the age when the mammoth and rhinoceros were native in Europe. The skulls are marked by the great thickness of the cranial bones, the size of the sinuses and the dolichocephalous conformation.

---

For the first time in the history of medicine yellow fever is found on the Pacific Coast of North America. There have been numerous cases in Western Mexico. In summer nearly the entire pacific Coast of the United States is suitable for the propagation of the disease and an epidemic there is feared.

---

Prof. Carrucio, of Modena, has discovered worms belonging to the ascarides family in hens eggs. Two specimens were exhibited by the writer before the Italian Academy of Medicine. The parasites were examples of the Heterakis inflexa.

---

Russia spends twenty-five per cent. more for the preservation of public health than for public education. "Public health is public wealth" is their motto.

---

Dr. Fernandez, of Barcelona, recommends the hypodermic injection of cobra poison for the cure of hydrophobia.

